# Profiling of *Petroselinum sativum* (mill.) fuss phytoconstituents and assessment of their biocompatibility, antioxidant, anti-aging, wound healing, and antibacterial activities

**DOI:** 10.3389/fnut.2024.1338482

**Published:** 2024-03-05

**Authors:** Ismail Mahdi, Paola Imbimbo, Hassan Annaz, Widad Ben Bakrim, Nihad Sahri, Asmae Alaoui, Daria Maria Monti, Mansour Sobeh

**Affiliations:** ^1^AgroBioSciences Program, College of Agriculture and Environmental Sciences, University Mohammed VI Polytechnic, Ben Guerir, Morocco; ^2^Department of Chemical Sciences, University of Napoli Federico II, Complesso Universitario Monte Sant’Angelo, Napoli, Italy; ^3^Physio-Chemical Laboratory of Inorganic and Organic Materials (LPCMIO), Materials Science Center (MSC), Ecole Normale Supérieure, Mohammed V University, Rabat, Morocco

**Keywords:** parsley, skin aging, oxidative stress, enzymatic activities, antibacterial activities, *Petroselinum sativum*

## Abstract

*Petroselinum sativum*, known as parsley, is a fragrant herb that possesses a rich heritage of utilization in traditional medicinal practices. In this study, we annotated the phytocontents of the aqueous and ethanolic extracts of *P. sativum* and investigated their antioxidant, cytoprotective, antiaging, wound healing, and antibacterial activities. LC–MS/MS analysis of both extracts revealed the presence of 47 compounds belonging to diverse groups including organic acids, phenolic acids, and flavonoids. By MTT assay, the extracts were fully biocompatible on immortalized human keratinocytes (HaCaT) while they inhibited intracellular ROS formation (DCFDA assay) and prevented GSH depletion (DTNB assay) upon UVA exposure. In addition, the extracts were potent in inhibiting the *in vitro* activities of skin-related enzymes mainly elastase, tyrosinase, collagenase and hyaluronidase. Using the scratch assay, *P. sativum* aqueous extract significantly enhanced wound closure when compared to untreated HaCaT cells. Moreover, both extracts inhibited *Pseudomonas aeruginosa*’s growth, reduced biofilm formation, and impaired the swimming and swarming motilities. Also, the aqueous extract was able to inhibit the production of bacterial pigments on plates. These findings strongly suggest the usefulness of *P. sativum* as a source of phytochemicals suitable for dermo-cosmeceutical applications.

## 1 Introduction

Aging is an inevitable process featured by impaired functions at the molecular, cellular, tissue and organ scales. It is the major risk factor for the development and progression of several diseases, especially in elderly subjects. Many diseases have been related to aging susceptibility such as diabetes, cardiovascular diseases, osteoporosis, neurodegenerative diseases, and cancer (1).

Over the past decades, many assumptions have been proposed to unveil aging process mechanisms. For instance, *in vitro* dysregulation of the apoptotic pathway was proven to be involved in some aging processes. Additionally, epigenomic changes greatly affect cellular function and profoundly contribute to aging progression (1). Also, scientific evidence has indicated the free radical reactions that occur within the cells and tissues, representing the major contributor to the aging process (2, 3). Indeed, understanding the underpinning mechanisms is critical for developing effective strategies against age-related diseases.

The cosmetic market is highly growing. In 2019, it generated estimated revenues of 380.2 billion USD and is expected to reach up to 463.5 billion USD by 2027 with a relative annual growth going from 5.3 to 7% between 2021 and 2027 (4). Consequently, bioactive compounds extracted from natural sources as cosmetic ingredients are gaining attractiveness. For instance, phytochemicals with antioxidant activities, modulatory effects on molecular cell receptors and/or gene expression, and inhibitory effects on enzymatic activity have demonstrated their usefulness in counteracting cell aging (5).

In this context, many functional foods and dietary compounds were found to have anti-aging properties and health beneficial effects *via* regulation of numerous pathways, such as nutrition sensing and stress signaling, modulation of cellular metabolism, stress resistance, energy homeostasis, genome maintenance, and mitochondrial biogenesis. Notwithstanding this evidence, many of these natural compounds are yet to be explored for their potential in preventing or delaying aging related disorders (6).

Parsley (*Petroselinum sativum*) is a biennial aromatic plant from the Apiaceae family. It contains flavonoids and other phenolic compounds such as furanocoumarins, carotenoids, polyacetylenes, and its leaves are a great source of minerals as calcium, thiamin, riboflavin, potassium and iron and vitamins such as A, C, and niacin (7). It harbors essential oil in all parts, with phenylpropane and terpene compounds as major constituents. *P. sativum* also contains proteins (2–22%), fats (4%), sugars, among others (8). Compounds such myristicin, apiol, 1-allyl-2,3,4,5-tetramethoxybenzene, β-phellandrene, 1,3,8-p-menthatriene, β-pinene, terpinolene, apiin, oxypeucedanin and falcarinol are mostly isolated from *P. sativum* (9). However, this composition depends on several factors, such as plant tissue, variety, climate, and sampling method (10).

For hundreds of years, Mediterranean population used *P. sativum* as a medicinal plant. Traditionally, it is used to manage arterial hypertension, diabetes, renal diseases, and cardiovascular diseases in different countries (11–13). Indeed, recent *in vitro* and *in vivo* studies demonstrated that *P. sativum* elicits several biological and pharmacological activities such as diuretic, anti-urolithiasis, hypouricemic, hypolipidemic, hypotensive, antioxidant, anti-inflammatory, and antiplatelet effects (9). It also exhibits antimicrobial, laxative, anti-anemic, anti-otitis, menorrhagic, antihyperlipidemic, cytoprotective, antihepatotoxic, carminative, estrogenic and hyperuricemia activities (11).

Although *P. sativum* has been explored for a wide range of biological activities, its cosmeceutical potential has not been sufficiently studied so far. Thereby, herein, we annotated the chemical composition of the aqueous and ethanolic extracts of *P. sativum* using LC–MS/MS and investigated their anti-aging potential, wound healing ability, as well as antioxidant activity using *in vitro*, enzymatic, and cell-based approaches. Moreover, the antibacterial, anti-biofilm and inhibitory effect on microbial mobilities of *Pseudomonas aeruginosa* were assessed. The choice of studying this bacterium resides in its multi-resistance to conventional antibiotics and its ability to cause skin infection (14). The overall aim of this study is to provide valuable information on the potential of *P. sativum* as a source of dermo-cosmeceutical agents, addressing critical aspects of skin care and dermatological health mainly anti-aging properties, protection against induced skin-oxidative stress, wound healing capabilities, and anti-infectious attributes.

## 2 Materials and methods

### 2.1 Plant material, extraction, phytocontents and LC–MS/MS analysis

Fresh *P. sativum* plants were obtained from a local market in Ben Guerir, Morocco, in December 2021. The aerial parts of the plants were shade-dried, milled into a fine powder, and then subjected to ultrasound-assisted extraction using water and/or ethanol (15 g × 400 mL) for 20 min, employing the following conditions: 20 Hz, 5°C, 30% sound wave amplification, and a 10-s pulse duration. Next, the extracts were filtered through glass-wool and the filtrates were centrifuged for 10 min at 6000xg to remove impurities. Next, the supernatants were evaporated using a rotavapor (BUCHI R-300, Flawil, Switzerland), frozen at −80°C and lyophilized (Labconco^™^, Strasbourg, France) to obtain completely dried fine powders. Extraction yields of the aqueous and ethanolic extract attained 1.71 g (11.4%) and 1.2 g (8%), respectively.

Total polyphenol (TPC) and flavonoids contents (TFC) were determined using the Folin–Ciocalteu and Aluminum chloride methods, respectively, as previously described (15). The LC–MS/MS analysis was conducted using a SHIMADZU LC MS 8050 coupled with a triple quadrupole spectrometer equipped with an ESI source (16). A C18 reversed-phase column (Zorbax Eclipse XDB-C18) with a flow rate of 1 mL/min and a gradient of water and acetonitrile (ACN) with 0.1% formic acid was employed for separation over 60 min. The autosampler (SIL-40C xs) facilitated automatic injection of 20 μL of the sample. Shimadzu’s LC solution software controlled the entire process, and the MS operated in the negative ion mode with an *m/z* range of 100 to 1,500. Compound identification relied on molecular weights, mass fragmentation patterns, and elution order compared to literature data from the plant and genus, as well as in-house standards.

### 2.2 *In vitro* antioxidant activities

The antioxidant activity of the extracts was estimated using the colorimetric methods, i.e., DPPH (2,2-diphenyl-1-picrylhydrazyl) and FRAP (ferric reducing antioxidant power) assays (17, 18). In the DPPH assay, 100 μL of the extract and 100 μL of the DPPH solution (0.2 mM) were combined in individual wells of a 96-well microplate and placed in darkness at room temperature for 30 min. The optical density (OD) was then measured at 517 nm. Quercetin served as the standard compound (ranging from 1 to 1,000 μg/mL). Each sample was tested in triplicate. The scavenging capacity was calculated as follows:

$$Scavenging\;effect\;\left(\%\right)=[Abs_{control}-Abs_{sample}/Abs_{control}]\times 100$$

The FRAP assay was also carried out in a 96-well plate. Briefly, 180 μL of the FRAP solution was mixed with 30 μL of the samples, shaken well, and kept in the dark at 37°C for 20 min. The distilled water served as the blank control and quercetin as the standard antioxidant. Ferrous sulfate (FeSO_4_) at the concentrations (500–7.81 μg/mL) was selected to draw a standard curve. All experiments were performed in triplicate. An intense navy-blue color was noticed upon reduction, which was monitored at OD_595 nm_ using a microplate reader. The results were expressed as mM FeSO_4_/g sample.

### 2.3 *In vitro* anti-aging activities

Anti-elastase, anti-tyrosinase, anti-collagenase and anti-hyaluronidase assays were performed as previously described (15). In brief, kojic acid and quercetin were used as positive controls, while solutions without samples were used as negative controls. Each measurement was performed in triplicate and the percentage of enzyme inhibition (%) was calculated according to the following formula:

$$\mathrm{Inhibition}\;\left(\%\right)=\left[\left(A_{\mathrm{control}}-A_{\mathrm{sample}}/A_{\mathrm{control}}\right)\times 100\right]$$

*A*_sample_: OD of the enzymatic activity from the extracts or positive control.

*A*_control_: OD of the enzymatic activity from the negative control.

### 2.4 Cell culture

Immortalized human keratinocytes (HaCaT, Innoprot, Spain) were cultured in Dulbecco’s Modified Eagle’s Medium (EuroClone), supplemented with 10% foetal bovine serum (HyClone), 2 mM l-glutamine and antibiotics (EuroClone) in a 5% CO_2_ humidified atmosphere at 37°C. HaCaT cells were cultured following the protocol described by Ferraro et al. (19).

### 2.5 MTT assay

Cells were seeded at a cell density of 2 × 10^4^ cells/cm^2^. After 24 h, increasing concentrations of each extract (from 0.1 to 100 μg/mL) were added to the cells for 48 h (20). Then, cell viability was evaluated by the MTT assay and results were expressed as cell viability rate (%) as described by Ferraro et al. (19).

### 2.6 UVA-mediated oxidative stress

Cells were seeded at a density of 2 × 10^4^ cells/cm^2^. After 24 h, cells were incubated for 2 h in the presence of 50 μg/mL of each extract and then exposed to UVA light (365 nm) for 10 min (100 J/cm^2^). A commercial UVA lamp was used as source of oxidative stress as described by Sobeh et al. (21).

The antioxidant effect of both aqueous and ethanolic extract was evaluated by measuring the intracellular ROS levels using DCFDA assay as described by Del Giudice et al. (22). Additionally, DTNB assay was used to analyze intracellular glutathione levels by following the method described by Petruk et al. (20). Commercial GSH was used as a standard to obtain a calibration curve.

### 2.7 *In vitro* wound healing scratch assay

The wound healing potential was determined utilizing a scratch assay. HaCaT cells were seeded at a cell density of 3 × 10^4^ cells/well and left for 24 h to reach 95% confluency. The cells were then washed using PBS, scratched manually using a 200 μL pipet tip, and incubated with 50 μg/mL of each extract. The healing progress of the scratch was observed at 0 h and 24 h *via* capturing images using an optical microscopy (Zeiss LSM 710, Zeiss, Germany) at 10x magnification. The wound’s width was assessed using Zen Lite 2.3 software. The findings are expressed as a measure of area reduction (fold change) compared to untreated cells.

### 2.8 Antibacterial activity

The antibacterial activity of *P. sativum* extracts was evaluated by the broth microdilution assay using a sterile 96-well microplate (23–27). A stock solution of each plant sample was suspended in Mueller Hinton (MH) broth, filtered through 0.22 μm sterile syringe filters, and two-fold serially diluted (100, 50, 25, 12.5, and 6.25 mg/mL). Subsequently, the diluted samples were dispensed into the wells of a microplate in triplicate (200 μL per well). Next, 2 μL of an 18-h-old bacterial culture of *P. aeruginosa* adjusted to OD_600nm_ = 1 were inoculated into each well and incubated at 37°C under 150 rpm shaking for 24 h. The lowest concentration that inhibited the visible microbial growth was considered as the minimum inhibitory concentration (MIC). The inhibition percentage of bacterial viability was calculated based on the OD value of the extracts treated media group against the extract free media.

### 2.9 Bacterial biofilm inhibition assay

The antibiofilm potential of the extracts was evaluated using a colorimetric assay (25–27). Firstly, the plant extracts with concentration of 12.5 mg/mL (1/8 maximum concentration) and 25 mg/mL (1/4 maximum concentration) were prepared in MH and then filtered using 0.22 μm sterile syringe filters. Filtered extracts were inoculated and incubated as described above. Media without bacterial cells addition were established as negative controls. Following 24 h incubation, culture media were removed, and wells were gently washed with PSB to remove planktonic bacterial cells. Then, the biofilm was stained with a crystal violet (CV) solution at 1% and incubated at room temperature for 15 min. Afterwards, a vigorous washing with sterile distilled water was performed to remove the excess of CV. The produced biofilm was dissolved by adding 95% ethanol to each well and the OD was read at 595 nm using a multimode plate reader (Costar, 96). The biofilm attachment was observed by using a microscope before and after solubilization by ethanol at 10x magnification.

### 2.10 Swimming and swarming inhibition assays

*P. sativum* extracts were tested for their effect on the swimming and swarming motilities of *P. aeruginosa* on agar plates. The swimming (1% tryptone, 0.5% sodium chloride, and 0.3% agar) and swarming (semisolid LB medium, 0.6%) media (28) were aseptically supplemented with 12.5 mg/mL and 25 mg/mL of each extract. Then, 5 μL of a fresh overnight culture of *P. aeruginosa* (OD_600nm_ = 1) was deposited at the center of each plate and incubated at 37°C for 24 h. The swimming and swarming zones diameters were measured in cm (15, 26). Fresh media were used as negative controls.

### 2.11 Statistical analysis

Statistical analysis was conducted using IBM SPSS Statistics 20 software, involving one-way analysis of variance (ANOVA) followed by a *post hoc* examination using Tukey’s test. Significance levels were considered at *p* < 0.05. Values are the mean of three independent experiments, each carried out in triplicate, and the outcomes are presented as the mean ± standard deviation.

## 3 Results

### 3.1 Phytochemical profiling

**Figure 1:**
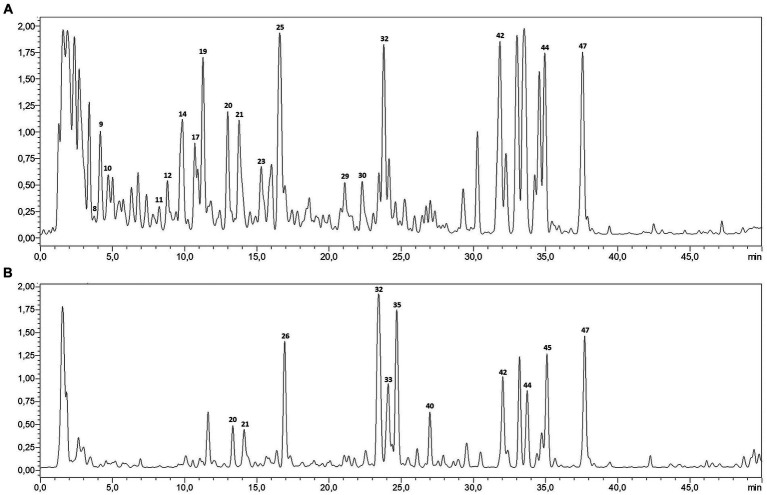
LC-MS profile of *P. sativum* aqueous extract **(A)** and ethanolic extract **(B)**.

The LC–MS/MS analysis of *P. sativum* aqueous and ethanolic extracts revealed a total of 46 phytocompounds with a variation in their content (Table 1; Figure 1). The aqueous extract contains 41 compounds, whereas 39 compounds were detected in the ethanolic extract. Notably, the aqueous extract contained the largest number and highest amounts of annotated compounds. These secondary metabolites belong to diverse groups, namely organic and phenolic acids, and flavonoids. Five signals, representing organic acids, were detected with high amount in both extracts, including oxoadipic, citric, maleic, malic, and succinic acids. In addition, the most abundant phenolic acid was coumaric acid, detected in its aglycon, glycosidic and acetylated conjugations.

**Table 1:** Annotated compounds from *P. sativum* extracts using LC–MS/MS.

No.	Rt (min)	[M-H] -	MS/MS	Proposed compounds	Aqueous extract	EtOH extract
1	1.20	159	115, 159	Oxoadipic acid	XXX	XXX
2	1.52	191	111, 191	Citric acid*	ND	XXX
3	1.81	115	115	Maleic acid	XXX	XXX
4	1.9	289	111, 115, 191	Citric acid malate	XXX	ND
5	1.975	133	115, 133	Malic acid*	XXX	XXX
6	2.67	173	111, 129, 173	Shikimic acid*	XXX	X
7	2.95	117	117	Succinic acid	XXX	X
8	3.53	331	125, 151	Gallic acid glucoside *	X	X
9	4.35	309	119, 163	Coumaric acid rhamnoside	XXX	X
10	6.36	431	137	hydroxybenzoic acid glucoside pentoside	X	X
11	8.16	315	109, 153	Protocatechuic acid glucoside*	X	X
12	8.70	285	108, 153	3,4-dihydroxybenzoic acid pentoside*	X	X
13	9.35	339	177	Esculetin glucoside*	XX	X
14	9.64	299	137	Hydroxybenzoic acid glucoside	XXX	X
15	10.62	323	101, 119, 162	Umbelliferon glucoside	XX	X
16	10.83	417	109, 153, 241	3,4-dihydroxybenzoic acid dipentoside	XX	X
17	10.95	341	135	Caffeic acid glucoside*	XX	ND
18	11.61	191	148, 176, 191	Scopoletin	X	ND
19	11.69	305	287, 305	Gallocatechin*	XXX	ND
20	12.67	457	119, 163, 357	Coumaric acid glucoside pentoside	XXX	XX
21	13.20	153	109, 153	3,4-dihydroxybenzoic acid*	XXX	XX
22	13.29	335	173	Caffeoyl shikimic acid	X	ND
23	15.17	593	353, 383, 473, 593	Isovitexin-8-C-glucoside (Vicenin 2)	XX	X
24	15.25	639	271, 301, 315	Isorhamnetin diglucoside	XX	X
25	16.41	325	119	Coumaric acid glucoside*	XXX	X
26	16.82	163	119, 163	Coumaric acid*	XXX	XXX
27	16.85	337	191	Coumaroylquinic acid	XX	XX
28	18.21	371	179, 209	Caffeoyl glucaric acid	X	X
29	21.78	651	285	Kaempferol acetylglucoside glucoside	X	ND
30	22.76	563	269	Apigenin apiosylglucoside	XX	X
31	23.26	271	151, 177	Naringenin	XX	XX
32	23.66	431	269	Apigenin glucoside	XXX	XXX
33	24.04	593	299	Diosmetin glucoside pentoside	XX	XX
34	24.19	595	301	Quercetin glucoside pentoside	ND	XX
35	24.44	477	271, 299, 301	Quercetin glucuronide	XX	XXX
36	25.22	463	301	Quercetin glucoside*	X	X
37	25.30	605	269	Apigenin apiosyl acetylglucoside	X	X
38	26.82	635	284, 299	Diosmetin apiosyl acetylglucoside	ND	X
39	26.90	649	269	Apigenin malonyl apiosylglucoside	ND	X
40	27.27	679	284, 299	Diosmetin acetyl glucoside glucuronide*	ND	XX
41	31.00	447	285	Kaempferol glucoside*	X	X
42	31.53	471	119, 163	Coumaric acid caffeoyl rhamnoside	XXX	XXX
43	32.19	557	119, 145, 163	Coumaric acid malonyl caffeoyl rhamnoside	XXX	XXX
44	34.65	269	117, 269	Apigenin	XXX	XXX
45	35.55	299	284, 299	Diosmetin	X	XXX
46	36.21	315	301, 315	Isorhamnetin *	X	X
47	38.26	513	119, 145, 163	Coumaric acid acetyl caffeoyl rhamnoside	X	XXX

Rt: Retention time, *in-house standards, ND: Not Detected, X: Minor, XX: Moderate, XXX: Major.

### 3.2 TPC, TFC and *in vitro* antioxidant activity

The analysis of polyphenol and flavonoid content revealed significant differences between the aqueous and ethanolic extracts. The ethanolic extract contained notably higher levels of both polyphenols (48.5 mg GA/g extract) and flavonoids (40.8 mg Quercetin/g extract) compared to the aqueous extract, which contained 29.44 mg GA/g extract of polyphenols and 6.55 mg Quercetin/g extract of flavonoids (Table 2). These findings suggest that the ethanol extraction method is more effective in extracting these bioactive compounds from the source material.

**Table 2:** Total polyphenol and flavonoids contents and *in vitro* antioxidant activities of *P. sativum* extracts.

Extracts	Total polyphenols (mg GA/g extract)	Total flavonoids (mg quercetin/g extract)	Antioxidant activity	
			DPPH (IC_50_, μg/mL)	FRAP (mM of FeSO_4_/g extract)
Aqueous extract	29.44 ± 0.20^b^	6.55 ± 0.20^b^	20.03 ± 0.30^a^	27.22 ± 0.50^b^
Ethanolic extract	48.5 ± 0.30^a^	40.8 ± 1.0^a^	25.76 ± 0.50^ab^	37.03 ± 0.20^a^
Quercetin	–	–	0.23 ± 0.01^d^	–
BHT	–	–	4.21 ± 0.08^c^	–

Letters in superscript indicate a statistical difference at *p* < 0.05.

In addition, the aqueous extract showed better DPPH scavenging activity with an IC_50_ of 20.03 μg/mL, while the ethanolic extract had a higher IC_50_ (25.76 μg/mL). However, in the FRAP assay, the ethanolic extract exhibited stronger ferric-reducing antioxidant power with a value of 37.03 mM of FeSO_4_/g extract compared to 27.22 mM of FeSO_4_/g extract for the aqueous extract. Reference compounds, quercetin and BHT, were significantly more active in DPPH scavenging activity compared to both extracts (Table 2).

### 3.3 Effect of *Petroselinum sativum* extracts on cellular viability

**Figure 2:**
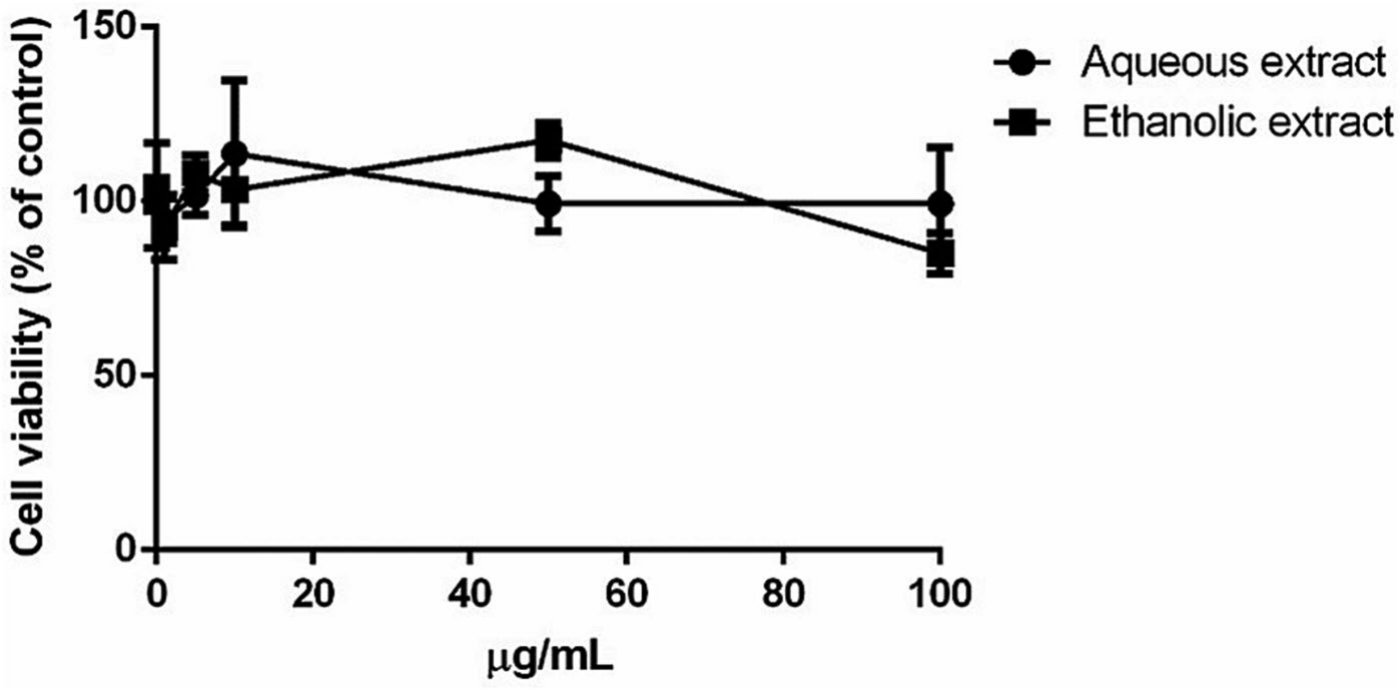
Dose–response curve of immortalized human keratinocyte (HaCaT) after 48 h incubation with increasing concentrations of *P. sativum* aqueous (black circles) and ethanolic (black squares) extract (0.1–100 μg/mL). Cell viability was determined using the MTT assay, and the percentage of surviving cells when exposed to test extracts, relative to control cells (those without extracts), was used to express cell survival. The data is presented as the mean values with standard deviation (S.D.), and three separate measurements were conducted.

The possible toxic effect of *P. sativum* extracts was evaluated on eukaryotic cells. HaCaT cells were incubated with increasing concentrations of each extract (0.1–100 μg/mL) for 48 h and then cell viability was evaluated utilizing MTT assay. Interestingly, no significant toxic effect on cell viability was observed in the presence of either extract. In particular, a complete biocompatibility was observed independently from the concentration tested (up to 100 μg/mL), suggesting that both extracts could be used safely on HaCaT cells (Figure 2). Starting from this encouraging result, the concentration of 50 μg/mL was used to study the protective activity of the extracts against UVA-induced oxidative stress.

### 3.4 Effect of *Petroselinum sativum* extracts on UVA-induced ROS formation and GSH-depletion

**Figure 3:**
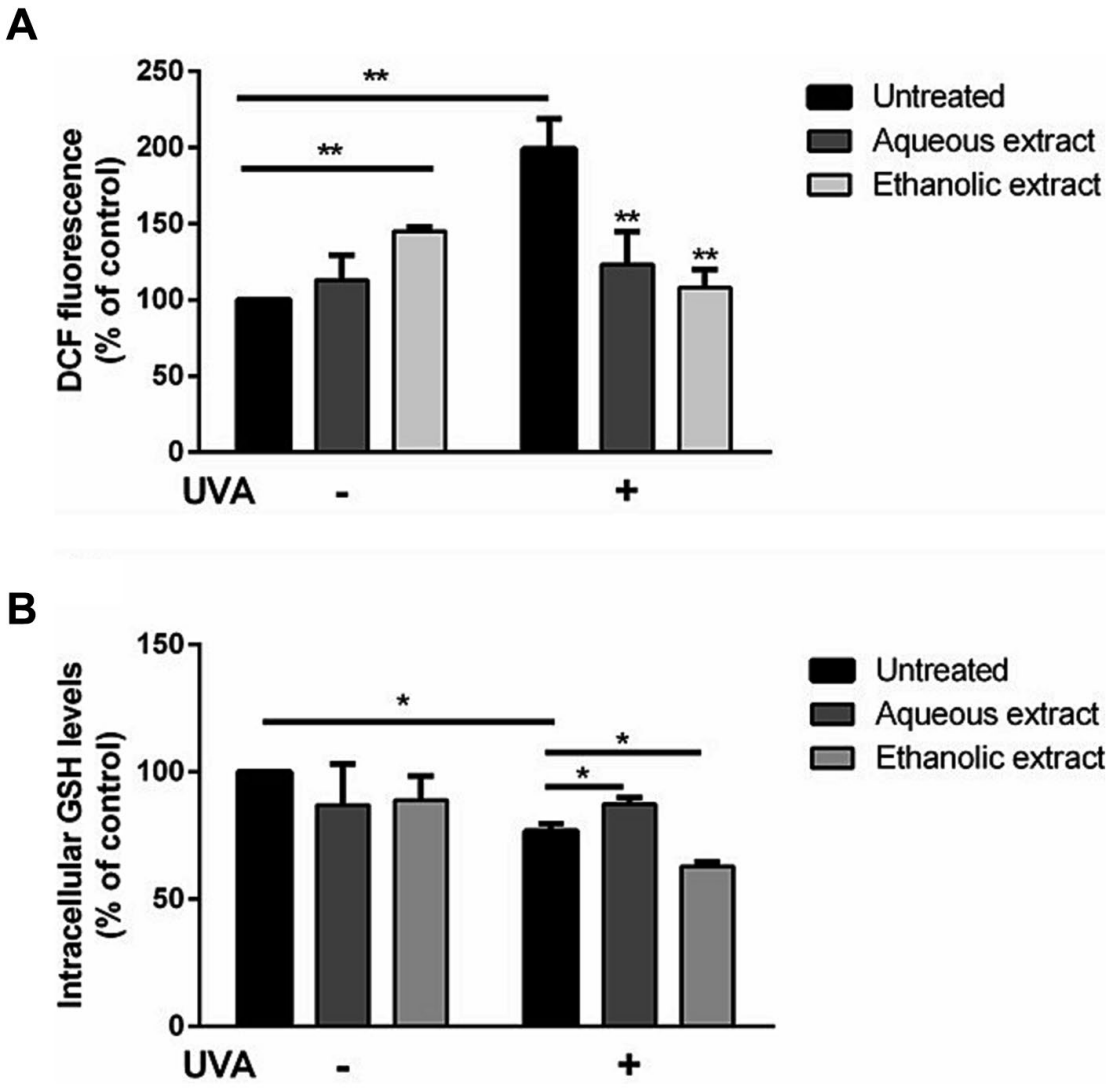
Antioxidant effect of *P. sativum* extracts on HaCaT cells stressed with UVA. Cells were pretreated with presence of 50 μg/mL of aqueous extract (dark grey bars) and ethanolic extract (light grey bars) for 2 h before UVA irradiation (100 J/cm^2^). Cells were cultured with extracts either without (−) or with (+) exposure to UVA. Black bars refer to untreated cells. **(A)** Determination of intracellular ROS levels utilizing DCFDA assay. **(B)** GSH levels measurement utilizing DTNB assay. The data are presented as percentages relative to untreated cells. The results represent the mean values with standard deviation (S.D.) from three independent experiments. * denotes significance at *p* < 0.05, while ** indicates significance at *p* < 0.01.

As shown in Figure 3A, ROS production was significantly increased after exposure to UVA irradiation (black bars, *p <* 0.01). Incubation of cells with the ethanolic extract (light grey bars) induced a significant increase in ROS production (*p* < 0.01) in the absence of oxidative stress. Interestingly, a significant inhibition of the intracellular ROS levels (*p* < 0.01) was observed when cells were pre-incubated with each extract.

As shown in Figure 3B, a slight but significant decrease in intracellular GSH levels was observed upon UVA irradiation (black bars). Interestingly, when cells pretreated with the aqueous extract before UVA exposure, GSH depletion was inhibited (up to 16.8% increase in GSH level), whereas a different behavior was observed when cells were pretreated with the ethanolic extract, as a significant depletion in GSH levels (up to 15% decrease in GSH level) were observed (*p* < 0.05).

### 3.5 Effect of *Petroselinum sativum* extracts on HaCaT cells migration

**Figure 4:**
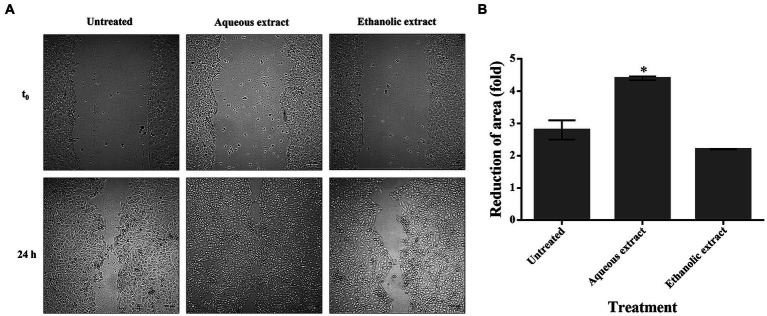
The effect of *P. sativum* extracts on wound healing. **(A)** Confluent HaCaT cells were scratched and treated with 50 μg/mL of either aqueous or ethanolic extract. At time 0 and after 24 h, optical microscopy images were acquired at 10x magnification and at different locations. **(B)** Reduction of area (fold) of wound closure upon 24 h incubation with aqueous or ethanolic extracts. The data are expressed as fold change relative to the control, which refers to untreated cells. The values represent the mean with standard deviation (S.D.) from three independent experiments. (*) signifies statistical significance at *p* < 0.05.

To verify if *P. sativum* extracts could promote cell migration in wound repairing, a scratch assay was conducted on HaCaT cells. In untreated cells, a spontaneous re-epithelialization was observed. Interestingly, the aqueous extract significantly enhanced the wound closure after 24 h of treatment (4.40-fold reduction) when compared to untreated cells (2.80-fold reduction). On the contrary, the ethanolic extract showed no positive effect, as wound closure was similar to untreated cells (Figure 4).

### 3.6 Effect of *Petroselinum sativum* extracts on cell aging-inducing enzymes

The enzymatic activities of both extracts were assessed against four the enzymes involved in cell aging namely elastase, tyrosinase, collagenase and hyaluronidase. The ethanolic extract (IC_50_ = 10.75 – 20.12 μg/mL) was more active in inhibiting the enzymatic activities than the aqueous extract (IC_50_ = 2.03 – 10.18 μg/mL). However, both extracts showed a higher inhibition of elastase and tyrosinase, compared to collagenase and hyaluronidase. Notably, the ethanolic extract exhibited pronounced inhibitory effect against the four enzymes, with respect to the reference compounds, i.e., Kojic acid and quercetin (IC_50_ = 9.00 – 24.83 μg/mL) (Table 3).

**Table 3:** *In vitro* inhibition activity of *P. sativum* extracts against skin aging-related enzymes.

Extracts		Elastase	Tyrosinase	Collagenase	Hyaluronidase
		IC_50_ (μg/mL)			
Aqueous extract		12.53 ± 0.3^b^	10.75 ± 1.9^a^	20.12 ± 1.1^a^	18.05 ± 2.1^a^
Ethanolic extract		3.0 ± 0.8^c^	2.03 ± 0.7^b^	10.18 ± 1.5^b^	12.54 ± 1.2^b^
Reference compound	Kojic acid	21.60 ± 0.9^a^	9.00 ± 0.9^a^	–	14.46 ± 0.6^ab^
	Quercetin	–	–	24.83 ± 1.8^a^	–

Letters in superscript indicate a statistical difference at *p* < 0.05.

### 3.7 Effect of *Petroselinum sativum* extracts on *Pseudomonas aeruginosa* growth, biofilm and mobilities

**Figure 5:**
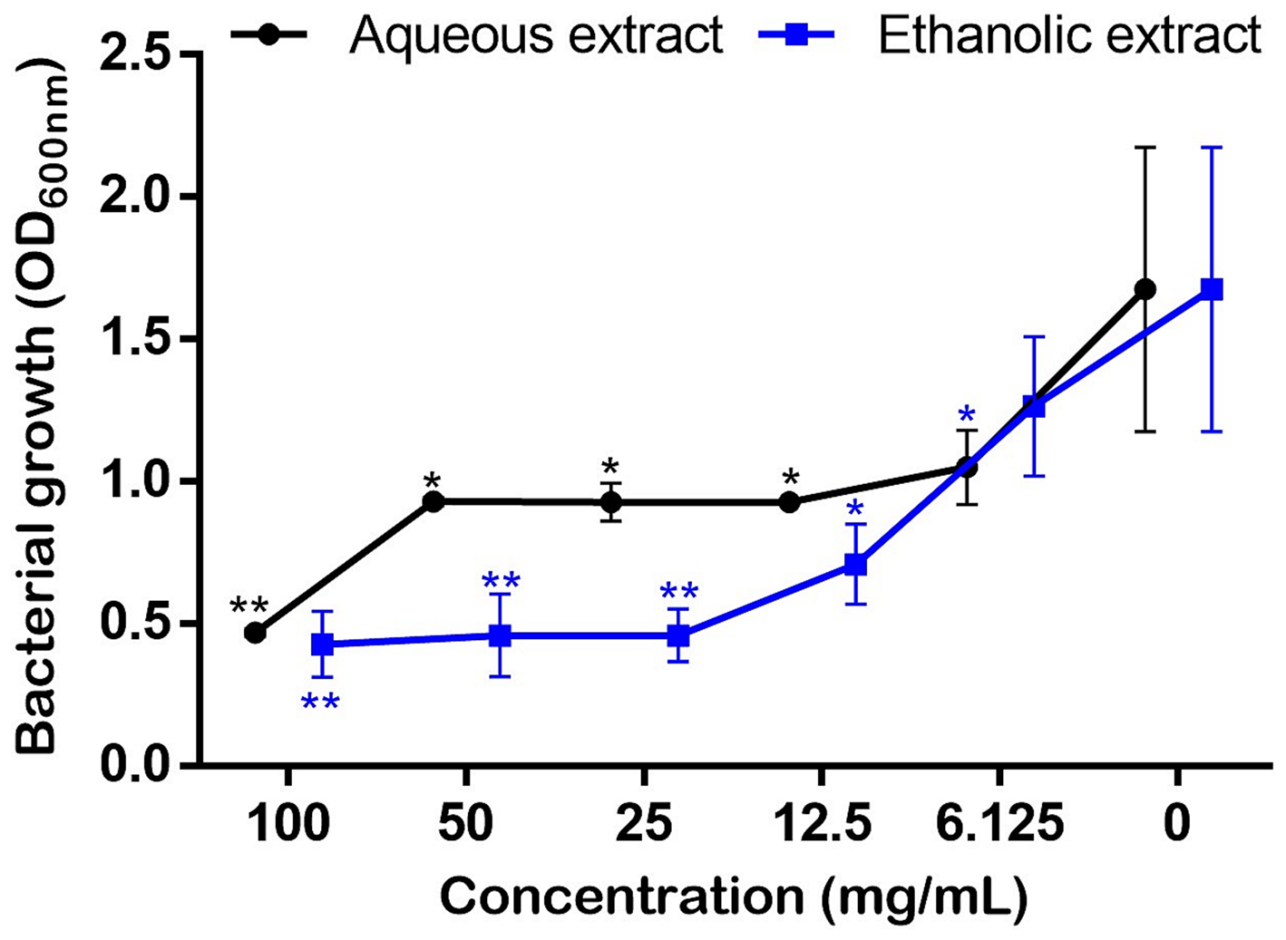
Effect of *P. sativum* extracts on the growth of *P. aeruginosa* using concentration rage 0–100 mg/mL in MH broth. Asterisks (*) indicate the statistical difference compared to the control media (*p* < 0.05).

Bacterial growth monitoring showed that the *P. sativum* extracts significantly and dose dependently inhibited *P. aeruginosa* growth starting from 6.125 mg/mL of the aqueous extract, and 12.5 mg/mL using the ethanolic extract. Nevertheless, even both extracts at 100 mg/mL decreased bacterial growth by 72.45 and 74.85%, respectively, total inhibition of bacterial growth did not occur (Figure 5). This means that the MIC is higher than 100 mg/mL.

**Figure 6:**
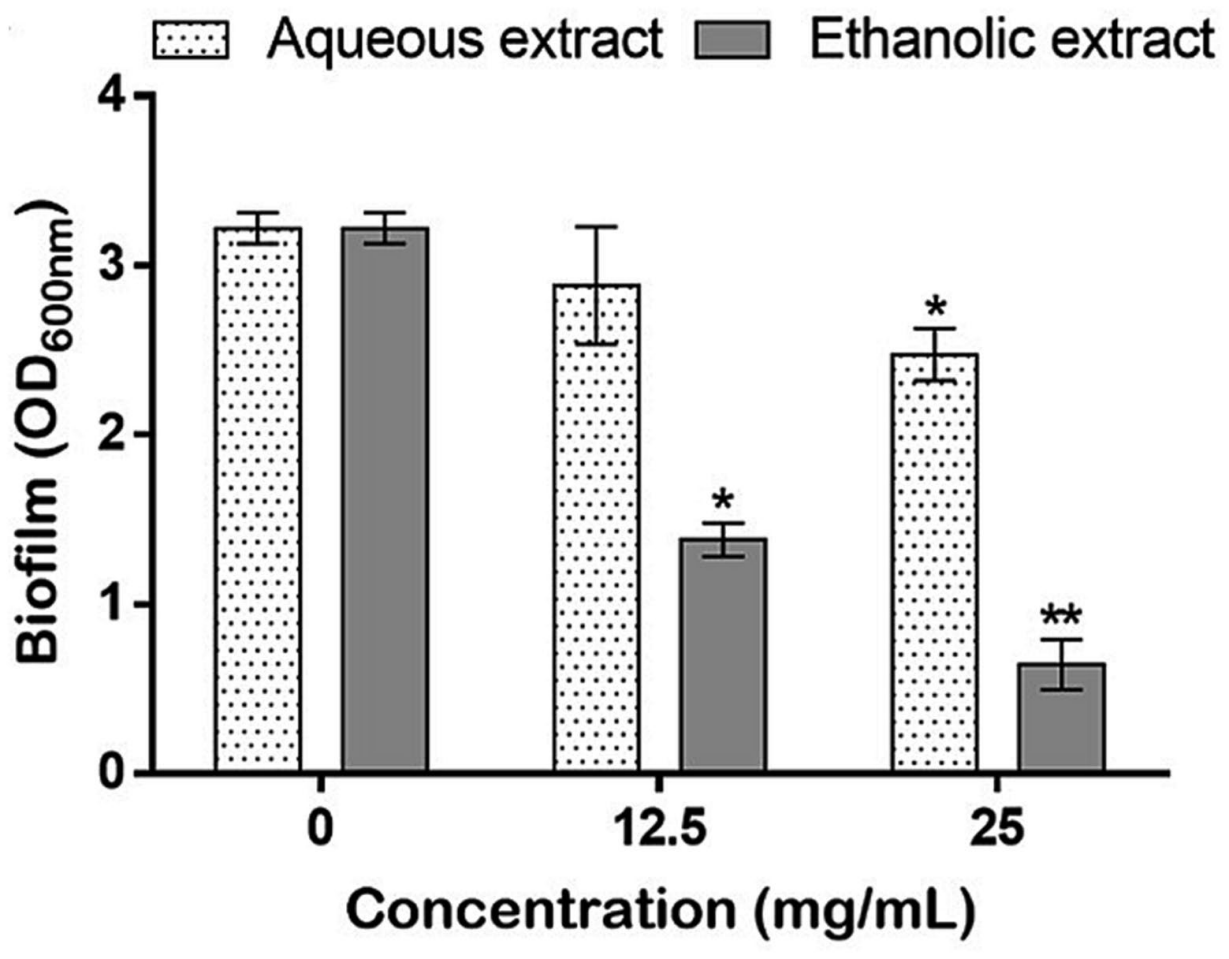
Effects of *P. sativum* extracts on the biofilm production by *P. aeruginosa* using the sub-doses of 12.5, and 25 mg/mL. Asterisks (*) indicate the statistical difference compared to the control media (untreated bacteria, 0 mg/mL) at *p* < 0.05.

To investigate the antibiofilm potential, the sub-doses of 12.5 and 25 mg/mL referring to 1/8 and 1/4 of the highest tested concentration (100 mg/mL), respectively, were utilized to estimate the amount of biofilm produced by *P. aeruginosa*. For both extracts, results showed a dose-dependent inhibition of the biofilm production, however the aqueous extract showed a slight, but significant, inhibition (23.05%) only at 25 mg/mL. On the other hand, the ethanol extract allowed a considerably inhibition in the biofilm formation at both tested concentrations (57% and 80%) (Figure 6).

**Figure 7:**
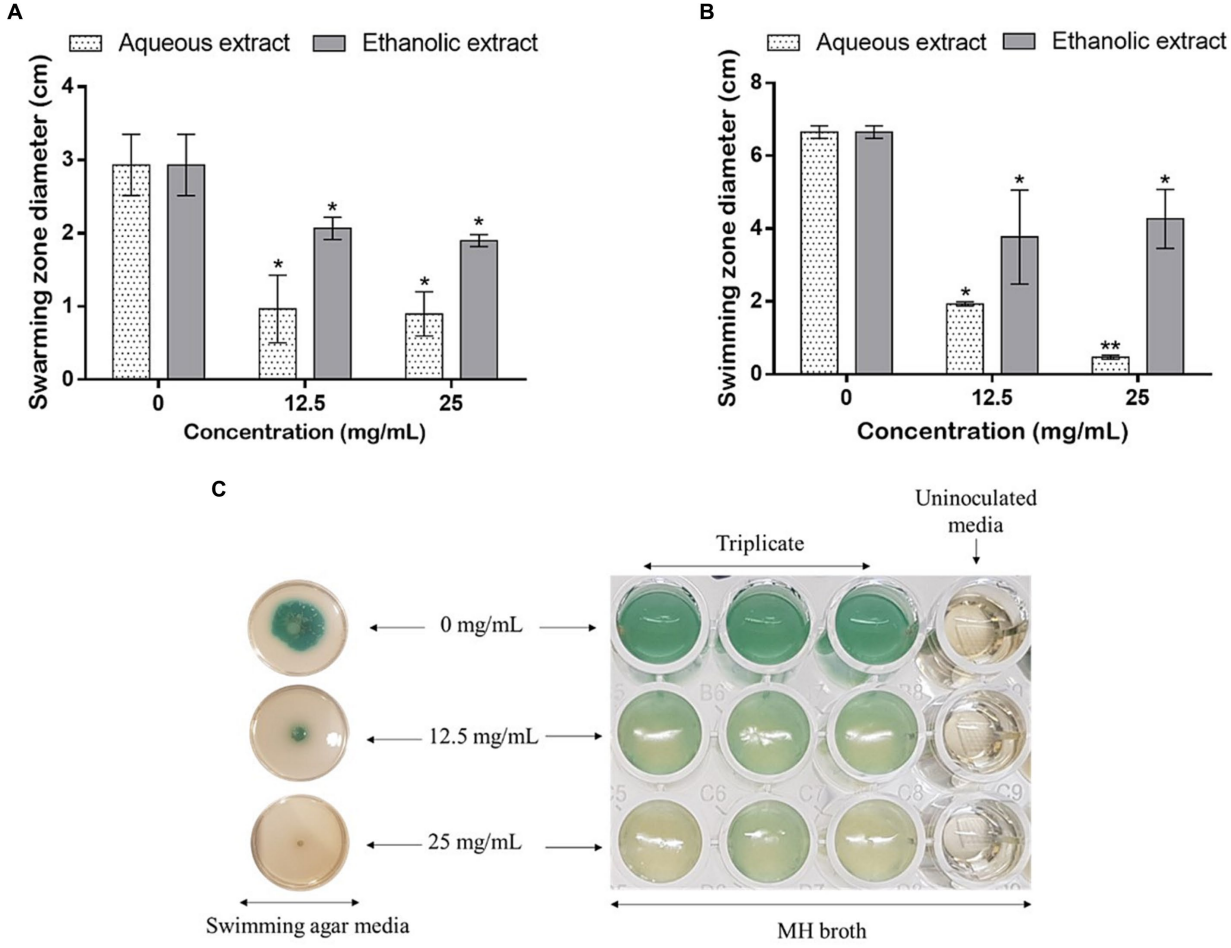
Effect of *P. sativum* extracts on **(A)** swarming and **(B,C)** swimming mobility of *P. aeruginosa* using the sub-doses 12.5 and 25 mg/mL. Asterisks (*) indicate the statistical difference compared to the control media (0 mg/mL) (*p* < 0.05).

Using the same concentrations, the effect of the extracts was tested on the mobility and bacterial pigment production on plates. As shown in Figure 7, the swarming (Figure 7A) and swimming (Figures 7B,C) mobilities were inhibited, as the concentrations of the extracts increased. Noteworthy, the water extract was more potent in hindering *P. aeruginosa* mobilities comparatively to the ethanolic one. Finally, the aqueous extract, at 25 mg/mL, completely inhibited the production of the bacterial pigments (Figure 7C).

## 4 Discussion

*P. sativum* is widely used as culinary herb, but it also boasts an array of potential health benefits that have been acknowledged over centuries. In this study, LC–MS/MS analysis of the aqueous and ethanolic extracts of *P. sativum* unveiled 47 secondary metabolites belonging to various classes of organic acids (e.g., oxoadipic acid, citric acid, maleic acid, malic acid, and succinic acid), phenolic acids (e.g., coumaric acid) and flavonoids (e.g., apigenin) (27, 29, 30). Similar findings were recently reported by Mallmann et al. (31) who identified 19 phenolic compounds in the methanolic extract (80%) of fresh *P. sativum* plants. The study showed that *P. sativum* is also rich in flavones, especially apigenin and its derivatives (31). Comparatively, the extracts obtained in the present study are rich in other phenolic acids, such as hydroxybenzoic acid, 3,4-dihydroxybenzoic acid and shikimic acid. Moreover, other aglycons were characterized along with their glycosidic and acetylated conjugations, such as isorhamnetin, diosmetin, quercetin, and kaempferol. In contrast, different types of polyphenols, mainly oleuropein, arbutin, myricetin, and naringin were recently revealed in the *P. sativum* hydro-ethanolic extract (32). The differences in the phytochemical composition are due to many factors, such as the geographical location, climate, soil type, growth stage, cultivation practices, and processing methods. These factors collectively shape the presence and concentration of the bioactive compounds, thus impacting therapeutic properties of the plant and its potential applications (10).

To the best of our knowledge, the cosmeceutical potential of *P. sativum* has not been sufficiently investigated. Thus, here we explored the beneficial effects of *P. sativum* aqueous and ethanolic extracts on skin aging process, wound healing, oxidative stress, and skin bacterial infections. Notably, the assessment of the extracts’ effect on HaCaT viability demonstrated a full biocompatibility for dermatological applications up to 100 μg/mL. More importantly, both extracts showed a strong *in vitro* antioxidant activity by DPPH and FRAP methods. These results were corroborated by antioxidant activity tested on a cell-based model, to evaluate the effects of *P. sativum* extracts on UVA stressed HaCaT cells where cell treatment with both extracts prior to UVA exposure resulted in a significant inhibition in intracellular ROS levels. The antioxidant activity of the aqueous extract was further confirmed by the inhibition of GSH depletion upon UVA radiation. Indeed, GSH is well known to be involved in the antioxidant defense system of the cells. However, a different behavior was observed for the ethanolic extract, as no protective effect was observed when cells were treated with the ethanolic extract and then irradiated by UVA radiations. Increased capacity of the aqueous *P. sativum* extract to prevent ROS and GSH depletion in UVA-irradiated HaCaT cells is probably due to the free radical scavenger activity of the plant phytochemicals (20). For instance, many preclinical investigations have provided evidence that apigenin, present in both extracts as a major compound, elicits positive impacts against UV-induced skin, dermatitis, vitiligo, wounds, skin aging, and skin cancer. This flavonoid primarily operates by impeding inflammation through the reduction of pro-inflammatory cytokines and internal inflammatory agents, alongside its antioxidative qualities that enhance the body’s natural defense mechanisms against oxidative stress (33).

Furthermore, *P. sativum* extracts were assessed for their capacity to inhibit skin aging-related enzymes. Skin aging is a complex process influenced by various factors, including enzymes like elastase, collagenase, hyaluronidase, and tyrosinase. Elastase and collagenase contribute to the breakdown of elastin and collagen fibers, crucial components necessary to maintain skin firmness and elasticity. Hyaluronidase degrades hyaluronic acid, a key molecule responsible for skin hydration and volume. Tyrosinase plays a role in melanin production, influencing pigmentation changes and age spots. The overactivity of these enzymes, coupled with natural reduction of repair mechanisms, results in an acceleration of skin aging by diminishing structural integrity, moisture retention, and even skin tone (34). Here, we showed that both extracts were potent for inhibiting *in vitro* enzymes’ activities to different extents. Interestingly, the ethanolic extract exhibited pronounced inhibitory effect (IC_50_ = 2.03–12.54 μg/mL) against the four enzymes comparatively the reference compounds, namely Kojic acid and quercetin (IC_50_ = 9.00–24.83 μg/mL). This could be explained by the synergistic action of the plant’s compounds to effectively imped the functioning of skin aging enzymes. Indeed, many of the identified phytoconstituents such as kaempferol, isorhamnetin, quercetin, and their analogous compounds have been previously shown to combat skin aging by exhibiting inhibitory effects against the aforementioned enzymes (34–37). The inhibitory impact of *P. sativum* extracts on skin enzymes is likely the result of multiple mechanisms. For instance, it has been shown that the essential zinc ion situated within the collagenase’s active site is pivotal for its operation, facilitating interaction with the inhibitor (38). Furthermore, the hydroxyl groups within polyphenols can engage with collagenase’s backbone or other functional side groups. Additionally, the hydrophobic interaction between the benzene ring in the polyphenolic compound’s structure and collagenase can induce conformational alterations, rendering the enzyme nonfunctional (39). Elsewhere, it was demonstrated that the application of *P. sativum* extract (50 μg/mL) to Human Dermal Fibroblasts (HDF) induced a significant decrease (up to 37.0%) in the protein expression levels of p-ERK and p-JNK, both known to be related to MMP1 gene expression by UVB exposure. These findings indicate that *P. sativum* extract potentially enhances wrinkle improvement by curbing the expression of the MMP1 gene, responsible for collagen breakdown. This effect is achieved through the hindrance of UVB-induced JNK phosphorylation (40).

Besides, a clinical trial provided evidence that the topical application of *P. sativum* resulted in a substantial reduction in the severity of epidermal melasma with comparable inhibitory effect to that of hydroquinone (41). Additionally, *P. sativum* leaf extract powder prepared at 4% was also reported to be effective in *in vivo* controlling of sebum. This effect was proposed to be attributed to the plant’s high vitamins content (C, A, B, E, K, and β-carotene) and minerals (magnesium, iron, phosphorus, manganese, sodium, potassium, sulfur, and calcium) (42, 43).

In this study, the effect of *P. sativum* extracts on HaCaT cells migration (*in vitro*) using the scratch assay was monitored and the aqueous extract significantly enhanced the wound closure (4.40-fold reduction), while no significant effect was observed with the ethanolic extract. Notably, this is the first study to demonstrate the *in vitro* wound healing effect of *P. sativum*. However, a recent study demonstrated that both the hydro-ethanolic and polyphenolic extracts of *P. sativum* resulted in substantial wound contraction in rats. After 25 days post-treatment period, the hydro-ethanolic extract exhibited the most pronounced healing effect, achieving up to 97% (32). This was attributed to the presence of bioactive compounds that facilitate repairing lesions and expediting cellular regeneration in impaired tissues. In fact, many medicinal plants (e.g., *Curcuma longa*, *Centella asiatica*, and *Paeonia suffruticosa*), along with their phytochemicals, are effectiveness in promoting wound healing. For instance, apigenin has been reported for its role in enhancing the wound healing process (44, 45). Furthermore, it has been reported that malic acid-enriched fractions from *Sempervivum tectorum* L. leaves promote cell proliferation and migration (46, 47). Therefore, it is plausible that the malic acid present in the *P. sativum* extracts may contribute to the wound healing process.

In addition to the skin aging and wound healing experiments, the antibacterial effects of *P. sativum* extracts against *P. aeruginosa* were investigated. The bacterium is an opportunistic pathogen that often displays multi-resistance to conventional antibiotics and is commonly associated to wound infections. In fact, when pathogens infiltrate a wound, they trigger an inflammatory response that can disrupt the orderly sequence of tissue repair. This leads to increased inflammation, delayed cell migration, impaired collagen synthesis, and compromised blood supply, all of which collectively hinder wound closure and tissue regeneration. Additionally, the presence of an infection can prolong the inflammatory phase, preventing the transition to subsequent healing stages (48, 49). Here, *P. sativum* extracts significantly and dose dependently inhibited *P. aeruginosa* growth. Precisely, the aqueous and ethanolic extracts at 100 mg/mL decreased bacterial growth by 72 and 75%, respectively. In addition, as *P. aeruginosa* can create robust biofilms, which sustain chronic infections, disrupt wound healing, and escalate the emergence of antibiotic resistance (50), the anti-biofilm capacity of the extracts was assessed and results revealed that the aqueous extract at 25 mg/mL significantly inhibited the biofilm up to 23% as well as the ethanol extract at 12.5 and 25 mg/mL which significantly decreased the biofilm by 57 and 80%, respectively. These results suggest that *P. sativum* phytocomponents hold potential for effectively contrasting *P. aeruginosa*’s biofilm formation during infections and thus impeding the adherence of bacterial communities to biological surfaces, in this case, the site of infection. In addition to biofilms, bacterial mobility assumes a crucial role in dictating the course of infection development. Bacteria employ various mechanisms such as flagella-driven twitching, swimming, or swarming to navigate the wound environment. These mobilities allow bacteria to colonize different wound surfaces and penetrate tissues (51, 52). The intricate interplay between bacterial mobility and the wound environment significantly impacts the severity, persistence, and treatment responsiveness of wound infections (53). Therefore, the effect of the extracts was assessed on the swarming and swimming mobilities of the bacterium on plates. Noteworthy, the aqueous extract was more potent in hindering *P. aeruginosa* mobilities comparatively to the ethanolic extract. At 25 mg/mL of the aqueous extract, the production of the bacterial pigment, pyocyanin, was completely inhibited. It is well established that *P. aeruginosa* only produces pyocyanin, therefore this is a diagnostic property of this bacterium. Pyocyanin, a redox-active phenazine compound, is a blue secondary metabolite found in sputum from *P. aeruginosa* infected tissues (54). This study demonstrated that *P. sativum* phytochemicals could target bacterial genes involved in biofilm formation and virulence factors production. Quercetin, a prominent constituent identified in the tested extracts, has previously demonstrated its efficacy in decreasing the expression levels of various quorum sensing genes namely lasR, lasI, rhlI, and rhlR exhibiting reductions of 68, 34, 57, and 50%, respectively (55). In the light of these findings, the potential of *P. sativum* phytoconstituents to counteract *P. aeruginosa* infections through its anti-quorum sensing effects holds promise through reducing bacterial growth rate, hindering biofilm formation and toxin production, and impeding bacterial colonization and infection within wound tissues.

## 5 Conclusion

Herein, *P. sativum* has been shown to be endowed with a wide range of cosmeceutical and dermatological properties. The phytochemical profiling of both extracts revealed the presence of a total of 47 phytocompounds belonging to diverse groups, namely organic and phenolic acids, and flavonoids. The plant was shown to be exempt of any cell toxicity, exhibited potent *in vitro* antioxidant activity, counteracted UVA-oxidative stress and GSH depletion in human keratinocytes, inhibited skin aging-related enzymes, enhanced wound healing process, inhibited the bacterial growth of *P. aeruginosa*, impaired biofilm formation, bacterial swimming and swarming, and the bacterial pigment (pyocyanin) production. These beneficial and protective effects are most likely due to the richness of the extracts in antioxidant compounds that enhance skin integrity, support collagen production, combat pathogens, and enhance the skin’s defense against oxidative stress. Nonetheless, additional investigations are imperative to uncover the complete capabilities of *P. sativum*, comprehend its modes of action and potential adverse effects on the skin, and develop efficient compositions to address diverse dermatological ailments.

## Data availability statement

The original contributions presented in the study are included in the article/supplementary material, further inquiries can be directed to the corresponding author.

## Ethics statement

Ethical approval was not required for the studies on humans in accordance with the local legislation and institutional requirements because only commercially available established cell lines were used. Ethical approval was not required for the studies on animals in accordance with the local legislation and institutional requirements because only commercially available established cell lines were used.

## Author contributions

IM: Investigation, Writing – original draft. PI: Investigation, Writing – original draft. HA: Investigation, Writing – original draft. WB: Investigation, Writing – original draft. NS: Investigation, Writing – original draft. AA: Writing – review & editing. DM: Investigation, Writing – review & editing. MS: Conceptualization, Validation, Writing – review & editing.
